# A question of balance: weighing the options for controlling ammonia, sulfur dioxide and nitrogen oxides

**DOI:** 10.1093/nsr/nwz088

**Published:** 2019-07-16

**Authors:** A R Ravishankara

**Affiliations:** Departments of Atmospheric Science and Chemistry, Colorado State University, USA

Acid precipitation, climate change and deteriorating air quality are some of the major environmental concerns of today, all caused by anthropogenic emissions of sulfur dioxide (SO_2_), carbon dioxide (CO_2_), hydrocarbons, nitrous oxide (N_2_O), nitrogen oxides (NO_x_), mercury and others. Societies have designed coping strategies by reducing specific emissions, depending on the environmental concerns of the times. The regulatory and management approaches for the different environmental issues have been formulated at different times. For example, acid precipitation was addressed as an environmental problem well before the anthropogenic climate change. In some countries, air-quality deterioration has been identified as an urgent problem only recently. Because of these reasons, there is incoherence in regulatory strategies and policies.

Acid precipitation is caused by emissions of SO_2_ and NO_x_ that are converted to sulfuric and nitric acids in the atmosphere. The acid precipitation leads to forest destruction and acidification of lakes and rivers that harm aquatic life. SO_2_ and NO_x_ also lead to the formation of particulate matter, which then influences climate change and degrades air quality. The particle formation is exacerbated if ammonia (NH_3_) is available by enhancing ammonium sulfate/ammonium bisulfate and ammonium nitrate aerosols—the visible air pollution. The formation of particulate matter with a size less than 2.5 micrometers, PM2.5, is particularly deleterious to human health. However, the enhanced ammonium reduces acid precipitation by neutralizing the acids before they are deposited, and thus mitigating acid precipitation.

**Figure 1:**
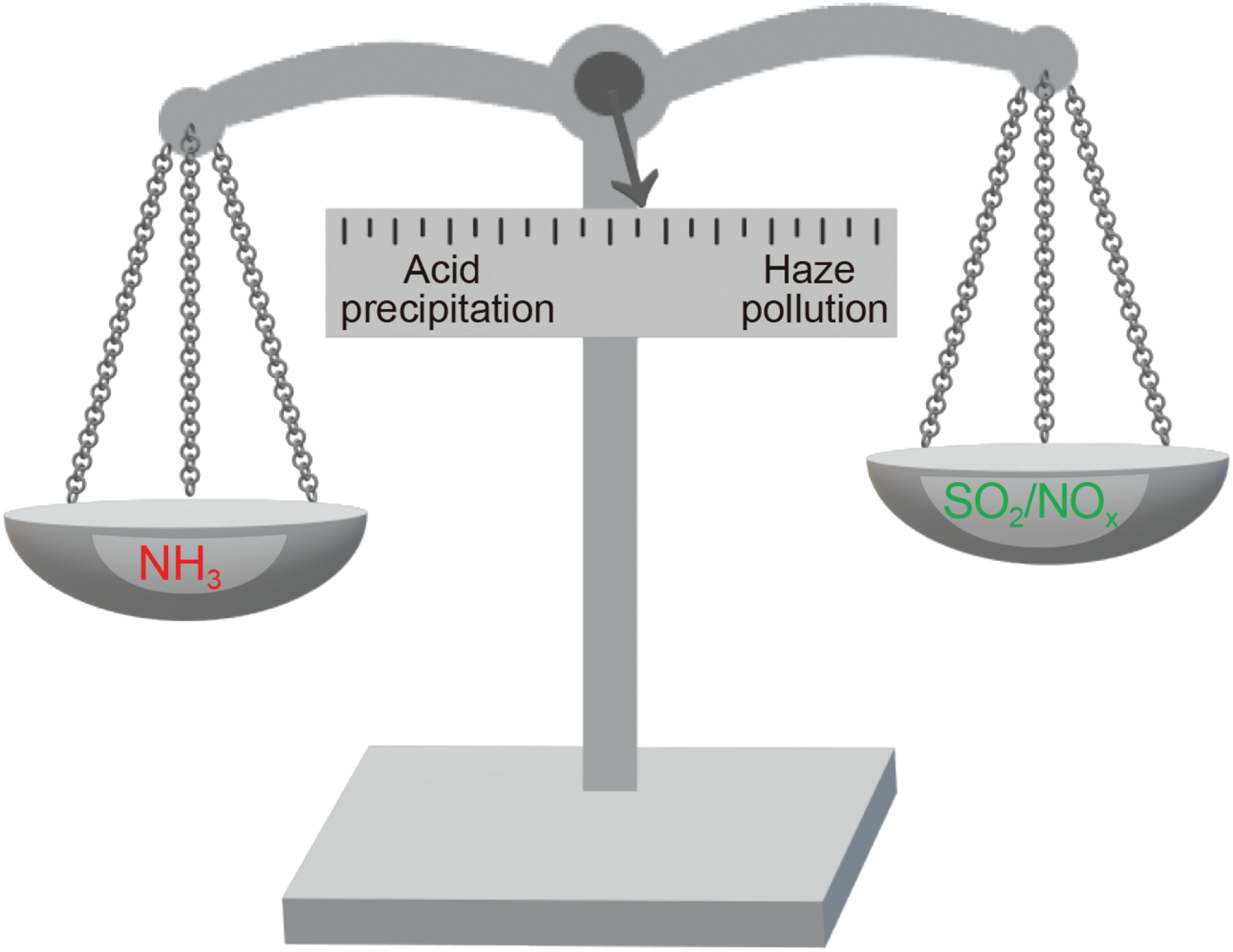
An artistic rendition of choices facing decision makers to control ammonia versus SO_2_/NO_x_ to simultaneously minimize haze pollution and acid precipitation.

The reasons for controlling ammonia and sulfur dioxide/nitrogen oxides can be different, and regulations are not always made with all the considerations in mind. This dilemma is shown by the paper by Liu *et al.* [1]. They show that this dilemma is different in different regions of China and in different seasons, highlighting potential decision-making complexity faced by environmental policymakers and managers. Weighing the benefits of NH_3_ versus SO_2_/NO_x_ control is indeed a balancing act (Fig. 1).

This balancing act gets more complicated if one were to consider other influences of these emissions and their origins. For example, NH_3_ is primarily due to food production (crops and animals), which also leads to emissions of N_2_O—a potent greenhouse gas and currently, the largest emission of an ozone-depleting gas [2]. Furthermore, fertilization and animal production lead to nitrogen deposition into soil and water bodies with degraded water runoffs. Therefore, a reduction in fertilization and controls on effluents from animal production would have additional benefits. However, such reductions will worsen acid precipitation, as noted by Liu *et al.* How should one weigh these issues? A holistic approach to environmental regulations would be beneficial. Such holistic assessments need better science, policy instruments and societal willingness.

One solution to reducing both acid precipitation and haze is to reduce SO_2_ emissions further. The emission of SO_2_ has already been reduced significantly [3] and, therefore, such a reduction may not be easy when weighed against the societal need for economic growth and increasing energy demands. Switching to sulfur-free fossil fuel would reduce SO_2_ emissions. This would not, however, reduce CO_2_, the most important human emission for climate change. The reduction of NOx, primarily from combustion, is another option. Switching to alternate energy sources such as solar and wind, on the other hand, would simultaneously reduce CO_2_, SO_2_ and NO_x_.

The second message from Liu *et al.* is that there is a clear connection between air quality and food production. The influence of air quality on food production has been explored a great deal [4], but not the influence of food production on air quality. Such influences can extend beyond the PM2.5 issue. In some regions, agriculture can also be a source of NO_x_ and volatile organics that influences the ozone. For example, methane, a significant part of what is emitted by food production (e.g. rice production and cattle- or sheep-raising), also increases global ozone levels and climate change. Indeed, there could be many other influences of agriculture on air quality, such as agricultural burning that produces smoke.

One necessity for handling this complex situation is evident: We need enhanced science to support well-informed and judicious policy-making.
